# Increased risk of proximal tubular dysfunction due to occupational cadmium exposure: a survival analysis study

**DOI:** 10.1093/joccuh/uiaf016

**Published:** 2025-03-05

**Authors:** Kento Hoshino, Satoko Iwasawa, Noriyuki Yoshioka, Satoko Suzuki, Itsumi Hashimoto, Shoko Ukita, Genta Toshima, Kengo Nagashima, Toru Takebayashi, Masashi Tsunoda

**Affiliations:** Department of Preventive Medicine and Public Health, National Defense Medical College, Saitama, Japan; Department of Preventive Medicine and Public Health, National Defense Medical College, Saitama, Japan; Department of Preventive Medicine and Public Health, National Defense Medical College, Saitama, Japan; Department of Preventive Medicine and Public Health, National Defense Medical College, Saitama, Japan; Department of Preventive Medicine and Public Health, National Defense Medical College, Saitama, Japan; Biostatistics Unit, Clinical and Translational Research Center, Keio University Hospital, Tokyo, Japan; Biostatistics Unit, Clinical and Translational Research Center, Keio University Hospital, Tokyo, Japan; Biostatistics Unit, Clinical and Translational Research Center, Keio University Hospital, Tokyo, Japan; Department of Preventive Medicine and Public Health, Keio University School of Medicine, Tokyo, Japan; Department of Preventive Medicine and Public Health, National Defense Medical College, Saitama, Japan

**Keywords:** cadmium, occupational exposure limit, renal dysfunction, proximal tubular dysfunction

## Abstract

**Objectives:**

The objective of the current study was to elucidate the relationship between blood cadmium (Cd-B) levels and proximal tubular dysfunction using urinary β2-microglobulin (B2M-U) as an indicator among workers in nickel-cadmium battery plants in Japan.

**Methods:**

Medical check-up data from 338 workers exposed to cadmium at 2 plants were collected from 1997 through 2020. Workers with at least 2 check-ups were included, excluding those with other renal dysfunctions. Proximal tubular dysfunction was defined as a B2M-U of 300 μg/g creatinine or higher in 2 or more consecutive check-ups. A multivariable Cox proportional-hazards regression model with time-dependent covariates was performed to analyze the relationship between Cd-B levels and the time to onset of proximal tubular dysfunction, adjusting for age, sex, and smoking history.

**Results:**

Of the 338 workers, 238 met the study eligibility criteria for the analyses. The geometric mean of Cd-B was 1.97 μg/L. The Cox proportional hazards analysis demonstrated that higher time-dependent Cd-B levels were significantly associated with an increased risk of proximal tubular dysfunction, with a hazard ratio of 1.17 (95% CI: 1.06-1.29).

**Conclusions:**

Higher Cd-B levels are associated with an increased risk of proximal tubular dysfunction in workers exposed to cadmium, indicating an increased risk of renal disease under the current industrial health management in Japan. Continuous monitoring and improved management of cadmium exposure are necessary to protect workers’ health even in developed countries such as Japan.

## Teaser

Key points

**What is already known on this topic:** Chronic cadmium exposures, even at levels lower than the exposure limits previously considered safe, have been reported to cause renal dysfunction, particularly in Europe. The EU's Scientific Committee on Occupational Exposure Limits has proposed lower, stricter, health-based biological limit values for cadmium.

**What this study adds:** We demonstrated that proximal tubular dysfunction occurs due to blood cadmium levels (Cd-B) among Japanese workers, even under strict exposure controls. Cd-B is a significant variable for the onset of proximal tubular dysfunction evaluated by Cox proportional hazards analysis, suggesting it could identify a population at higher risk. In addition, the analysis using the cut-off value for Cd-B of 5 μg/L—the occupational exposure limit in Japan—revealed a 2.4-fold increased risk of disease development, although not statistically significant.

**How this study might affect research, practice, or policy:** The higher Cd-B levels observed among present-day Japanese workers were positively associated with an increased risk of developing proximal tubular dysfunction. The significant risk of proximal tubular dysfunction among workers under Japan's current occupational management standards warrants careful reconsideration, focusing on the need to discuss and re-evaluate proper exposure standards.

## 1. Introduction

Cadmium (Cd) is one of the most cumulative and toxic metals that humans can be exposed to in an occupational environment.^1^ A study has shown that the incidence of serious poisoning caused by occupational Cd exposure has declined worldwide over the past 4 decades.^2^ This decrease is likely a result of proper management and education in developed countries. Remarkably, comprehensive education and changes in the sanitation program of a certain workplace were able to cut Cd levels by 50%.^3^

However, based on recent studies, renal effects have been observed in the general European population (mainly exposed by the oral route) at levels even below 2 μg Cd/g creatinine (gCr) in urine.^4^ Therefore, stricter standards for Cd exposure have been proposed. The German Research Foundation revised the Biologischer Arbeitsstoff-Referenzwert for blood cadmium (Cd-B) to 1 μg/L.^5^ For the limit of occupational Cd exposure, the Scientific Committee on Occupational Exposure Limits in the EU has re-evaluated the levels for Cd and proposed health-based biological limit values (BLVs) for urinary cadmium (Cd-U) of 1 μg/gCr.^6^ We have reached a critical point whereby we need to re-evaluate the current standards for Cd in occupational exposures to determine whether or not stricter standards are necessary in developed countries, including Japan.

The Japanese Society for Occupational Health (JSOH) has set the occupational exposure limit for Cd-B at 5 μg/L.^7^ To re-evaluate this value, a study using data from Japanese workers exposed to cadmium was necessary. Furthermore, because occupational exposure is long term, it was necessary to use data from long-term exposures. However, to date, there have been few studies of long-term occupational Cd exposure and the related health effects,^8^ particularly studies using Japanese data. In collaboration with Keio University, we collected data from 1997 through 2024 from workers' medical check-ups at 2 cadmium-exposed plants in Japan considered appropriate to analyze long-term occupational Cd exposure and the related health effects.

The earliest health effects of Cd are renal dysfunctions, especially proximal tubular dysfunction, which results in increased urinary excretion of low molecular weight proteins such as urinary β2-microglobulin (B2M-U).^9^ The reference value for proximal tubular dysfunction is controversial, but in previous reports approximately B2M-U ≥ 300 μg/gCr has been used.^8^^,^^10^^,^^11^ Renal dysfunction due to mild elevations above 300 μg/gCr is reversible and normalizes if exposure is discontinued.^11^ However, as occupational exposure to Cd is ongoing, even a slight elevation above 300 μg/gCr is considered irreversible and is the threshold for proximal tubular dysfunction.

To re-evaluate the current standard, it is important to clarify the relationship between long-term Cd exposure and proximal tubular dysfunction, an early indicator of renal dysfunction. The relationship between Cd-B and B2M-U has been evaluated in previous studies.^12^^,^^13^ However, these were cross-sectional designs. To evaluate long-term Cd exposure, the Cd-B levels every 6 months, based on repeated Cd measurements, were analyzed. In the present study, we evaluated whether or not Cd-B levels at respective medical check-ups affect the occurrence of proximal tubular dysfunction using survival time analysis from 1997 through 2020. The objective of the present study was to elucidate whether or not Cd exposure causes proximal tubular dysfunction under the current industrial health control limits in Japan. The assessment of Cd-B in the long term would contribute to the evaluation of the current state of occupational environments and Cd exposure management in Japan.

## 2. Methods

### 2.1. Sample collection and data definition

The participants in this study were workers from 2 nickel-cadmium (Ni-Cd) battery plants in Japan that use Cd sulfate, Cd oxide, and Cd hydroxide to produce Ni-Cd rechargeable batteries. The air Cd concentrations in these plants were controlled and maintained to below 0.05 mg/m^3^ by strict industrial management. However, even under such low Cd levels in the air, workers could likely have been exposed to Cd from inhalation of dust, fumes, and/or mist containing Cd.

Between 1997 and 2020, a total of 338 workers (232 males, 102 females, and 4 unknown) underwent periodic medical check-ups for Cd exposure as required by the Ordinance on Prevention of Hazards Due to Specified Chemical Substances. The cohort included administrative staff not always engaged in Cd-related work. Workers who had undergone at least 2 medical check-ups were included in the study, whereas those with a history of renal dysfunction were excluded.

Background information, such as date of birth and smoking history, was obtained from the questionnaire at the medical check-ups. Age was calculated based on the date of birth and the date of the medical check-ups. On each questionnaire, participants were asked, “Do you smoke?” A participant's response of "yes" at least once was categorized as that participant having a history of smoking. A participant's follow-up ended when at least 1 of the following occurred: cessation of medical check-ups, data censoring if the participant met the criteria for proximal tubular dysfunction during the study period, or the end of the study period. The cessation of medical check-ups was defined as not having undergone a medical check-up for longer than 12 months, in which case the day of the last medical check-up before cessation was considered the endpoint. Records missing Cd-B data on the day of check-ups were treated as missing values and excluded.

### 2.2. Determination of Cd-B and B2M-U

The Cd-B levels in the samples collected in the check-ups were determined using a Z-2710/Z-5010 Atomic Absorption Spectrophotometer (Hitachi High-Tech Fielding, Tokyo, Japan). The lower limit of quantification (LLOQ) was the concentration equivalent to the Cd signal that was equal to 10 times the SD of 10 repeated measurements of a blank signal, and the LLOQ of Cd-B was 1.00 μg/L. B2M-U was assessed using the latex agglutination method by SRL, Inc. (Tokyo, Japan). The value of the detection limit of B2M-U was 10.0 μg/L. Values below the LLOQ or the detection limit were treated by imputing LLOQ/2. B2M-U levels corrected with urine creatinine (B2M-U [μg/gCr]) were used for all the analyses. No imputations were applied to B2M-U values, as the analysis was based on a binary classification using the threshold of 300 μg/gCr for proximal tubular dysfunction.

### 2.3. Statistical analyses

All 238 eligible workers were included in the analyses. Descriptive statistics characterized the study population. A multivariate Cox proportional-hazards regression model with time-dependent covariates was used. Time zero corresponded to the initiation of medical check-ups for each worker. The objective variable was proximal tubular dysfunction, and the explanatory variables were: Cd-B as a time-dependent covariate, age, sex, and smoking history. Proximal tubular dysfunction was defined as a B2M-U of 300 μg/gCr or higher in 2 or more consecutive medical check-ups. To exclude transient elevations due to inflammation or other factors,^14^ the criterion of 2 consecutive high values was used to account for the irreversibility of cadmium-induced renal damage. Because Cd-B levels change over time, values measured repeatedly every 6 months were used as time-dependent covariates. Two types of analyses were conducted. The first analysis treated Cd-B as a continuous variable. The second analysis used a binary classification of Cd-B with a cut-off of 5 μg/L.

R statistical software, version 4.3.2 (R Foundation, Vienna, Austria), was used for the multivariate survival analyses with time-dependent covariates. SPSS software, version 28 (IBM, Armonk, NY, USA), was used for all other statistical analyses.

### 2.4. Ethical approval

This study was approved by the Ethics Committee of the National Defense Medical College (approval no. 4684). Participants were informed about the study by posts on the National Defense Medical College website and could opt out at any time. The study was conducted following the principles of the 2024 version of the Declaration of Helsinki.

## 3. Results

**Figure 1:**
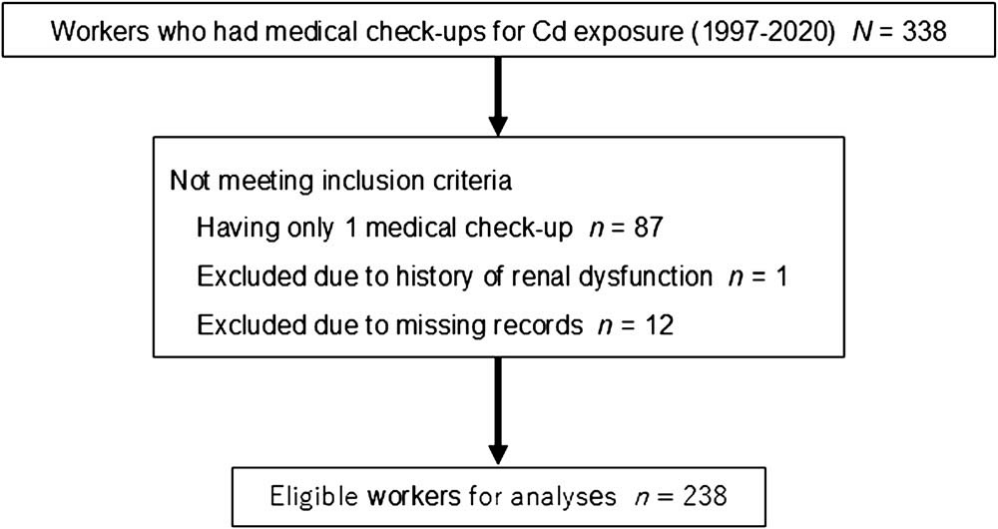
Flowchart of participant selection for study analyses.

Figure 1 shows the flowchart for selecting participants for the study analyses. Of 338 workers, 251 who underwent at least 2 medical check-ups were included in the study. One worker was excluded due to a history of a renal dysfunction, and 12 were excluded due to missing blood data for Cd-B. After these exclusions, 238 workers met the study eligibility criteria. Table 1 shows the participants’ characteristics at baseline. Their mean age was 34.5 years, and 31.1% were women. Half of the participants had a history of smoking. The mean (SD) person-years of follow-up per participant was calculated as 7.70 (7.01) years. A total of 3568 Cd-B measurements from the 238 participants were included in the analyses. Table 2 shows the geometric mean (GM) of Cd-B among the participants as 1.97 μg/L, and 9.7% of the measurements had a Cd-B ≥5 μg/L. Of the total Cd-B measurements, 13.2% were below the LLOQ and were treated as LLOQ/2.

**Table 1:** Characteristics of the participants at baseline.^a^

**Characteristics**	**Eligible participants (*n* = 238)**
Age at entry, mean (SD), y	34.5 (10.6)
Female sex, *n* (%)	74 (31.1)
Smoking history, *n* (%)	123 (51.7)
Proximal tubular dysfunction, *n* (%)	30 (12.6)
Total person-years of follow-up, y	1831.5
Per participant, mean (SD), y	7.70 (7.01)
No. of measurements, mean (SD), times	15.0 (12.8)

a Mean age at entry, total person-years of follow-up, person-years of follow-up, and number of measurements = arithmetic means.

**Table 2:** Characteristics of Cd-B among the participants at the check-ups.

**Characteristics of Cd-B**	**Eligible measurements (*N* = 3568)**
Geometric mean (GSD), μg/L	1.97 (2.13)
Imputing LLOQ/2, *n* (%)	472 (13.2)
Cd-B_5 (Cd-B ≥ 5 μg/L), *n* (%)	346 (9.7)

Abbreviations: Cd-B, blood cadmium; GSD, geometric standard deviation; LLOQ, lower limit of quantification.

Table 3 shows the results of the first analysis with Cd-B as a continuous variable named “time-dependent Cd-B.” According to the Cox proportional hazards model, which was used to obtain adjusted hazard ratios for each covariate, including “age at entry,” “sex,” “smoking history,” and “time-dependent Cd-B,” related to proximal tubular dysfunction, the hazard ratio for “time-dependent Cd-B” was 1.17 (95% CI: 1.06-1.29).

**Table 3:** The HR for proximal tubular dysfunction for the participants by Cox proportional hazards model: Cd-B as a continuous variable.

**Variable**	**HR**	**95% CI**	***P* value**
Age at entry	1.05^*^	1.01-1.09	.03
Sex	0.76	0.32-1.81	.54
Smoking history	0.65	0.31-1.38	.27
Time-dependent Cd-B	1.17^*^	1.06-1.29	.002

Abbreviations: Cd-B, blood cadmium; HR, hazard ratio.

^*^ *P* < .05.

The results of the second analysis with Cd-B as a binary classification are presented in Table 4. When the “time-dependent Cd-B” was dichotomized with a cutoff value of 5 μg/L, the hazard ratio was 2.40 (95% CI: 0.92-6.24), which did not reach the level of statistical significance (*P* < .07).

**Table 4:** The HR for proximal tubular dysfunction among the participants by Cox proportional hazards model: Cd-B as a binary classification.

**Variable**	**HR**	**95% CI**	***P* value**
Age at entry	1.05^*^	1.01-1.10	.02
Sex	0.76	0.32-1.81	.54
Smoking history	0.66	0.31-1.40	.27
Cd-B_5 (Cd-B ≥ 5 μg/L)	2.40	1.92-6.24	.07

Abbreviations: Cd-B, blood cadmium; HR, hazard ratio.

^*^ *P* < .05.

## 4. Discussion

In this study, we analyzed the medical check-up data from workers exposed to Cd in Japan using the Cox proportional hazards model to elucidate the relationship between Cd-B levels and the survival time of proximal tubular dysfunction. Cd-B is measured as an indicator of Cd exposure in Japan, and B2M-U is measured as an indicator of its health effects. Stricter standards for Cd exposure have been proposed, mainly in European countries, and it remains unclear whether or not kidney damage, especially proximal tubular dysfunction, has occurred under Japan's current industrial management. Therefore, by examining the relationship between repeated measurements of Cd-B and B2M-U, we aimed to confirm the relationship between Cd exposure and B2M-U under current industrial management conditions in Japan and to verify the appropriateness of the standard values.

The overall GM of Cd-B was 1.97 μg/L. The median of Cd-B in the general Japanese population was reported as 1.00 μg/L in 2017.^15^ Median Cd-B in pregnant women in Japan was reported as 0.70 μg/L in 2022.^16^ It is suggested that the workers in the present study had higher Cd burdens due to occupational exposure. In addition to occupational exposure, the factors contributing to the higher GM of Cd-B were higher smoking rates and a larger proportion of male participants in the present study compared with the general population. According to the National Health and Nutrition Survey in Japan, the adult smoking rate in 1997 was 52.7% for men and 11.6% for women.^17^ However, there has been a general downward trend since then; the adult smoking rate in 2022 was 24.8% for men and 6.2% for women. Based on these data, it could be inferred that the smoking rate among the study participants was higher than that of the general population.

Several factors, including age and smoking status, were adjusted for in the analyses for the relation between Cd-B and B2M-U due to their correlation with Cd-B levels.^1^^,^^18^^,^^19^ Previous studies have shown that Cd-B levels can be up to 4 or 5 times higher in smokers compared with those in nonsmokers,^18^ and that Cd levels increase with age up to 60-70 years.^1^ Additionally, women of reproductive age tend to absorb more Cd from their diet than do men.^19^ Therefore, in addition to Cd-B, we included sex, age, and smoking history as variables in our Cox proportional hazards model.

The threshold for proximal tubular dysfunction was set at 300 μg/gCr based on previous studies^10^^,^^11^ that have used this value as a benchmark. This threshold has also been reported as an independent risk factor for a rapid decline in the estimated glomerular filtration rate among the Japanese population.^20^ In the dataset for the present study, 30 workers (12.6%) had proximal tubular dysfunction, suggesting potential renal impairment.

The results of the Cox proportional hazards analysis, with Cd-B as a continuous variable, revealed that time-dependent Cd-B had a significantly higher hazard ratio, 1.17 (95% CI: 1.06-1.29), for proximal tubular dysfunction after adjusting for sex, age, and smoking history. The higher Cd-B levels observed among the Japanese workers in the present study were associated with an increased risk of developing proximal tubular dysfunction, as measured by B2M-U, which could identify a population at higher risk.

When using a cutoff value of 5 μg/L for time-dependent Cd-B, the hazard ratio was 2.40 (95% CI: 0.92-6.24). Although this was not statistically significant, the threshold value indicates a 2.4-fold increased risk of disease development. When Cd-B exceeds the cutoff value of 5 μg/L, which is equal to the values recommended by the JSOH^7^ and the American Conference of Governmental Industrial Hygienists,^21^ an increased risk of proximal tubular dysfunction may occur. Continuing to periodically evaluate the validity of management standards is critical to the health of workers in Ni-Cd battery plants in Japan.

Age at entry also showed a significantly higher odds ratio, indicating that both higher Cd-B levels and older age at entry are associated with a higher risk of developing proximal tubular dysfunction per unit time. For age as a significant variable, the following mechanism has been suggested. In normal kidney tubular epithelial cells, α-Klotho, an anti-aging protein, is highly expressed.^22^ However, it has been reported that α-Klotho levels decrease in the elderly.^22^ Consequently, the elderly are more prone to tubular dysfunction due to reduced tubular function. This finding is consistent with the results of the present study.

Regarding smoking, the hazard ratio for proximal tubular dysfunction was 0.65 (95% CI: 0.31-1.38). This study may have underestimated the effect of smoking because, due to data collection, smoking status was determined based on past history rather than current smoking behavior.

There are possible limitations to this study. First, we did not obtain information on precise Cd levels in the air in the work environment. However, Cd-B levels serve as a reliable indicator of Cd exposure. Second, it remains unclear which contributes more to a Cd burden, dietary or occupational exposure sources. However, dietary exposure to Cd is generally decreasing.^23^ It is plausible to consider that higher levels of Cd-B are mainly due to occupational exposure. Third, the interaction term between sex and Cd-B was not statistically significant in our analyses, and thus we did not perform separate analyses by sex. However, the possibility of sex-specific differences cannot be entirely excluded. Future studies with larger datasets should consider sex-specific analyses to further investigate potential sex differences in Cd exposure and proximal tubular dysfunction. Finally, there are potential limitations related to the measurement method and data interpretation. The LLOQ for Cd-B in this study was 1 μg/L, which is relatively high compared to the GM of Cd-B (2.0 μg/L). This may have influenced the distribution of measured values, particularly among individuals with lower Cd burdens. Additionally, measurements below the LLOQ were imputed as LLOQ/2, which could introduce bias by underestimating Cd-B levels for individuals with lower exposures. The Cd-B analysis was conducted at the Department of Hygiene and Public Health, Keio University School of Medicine. The spike recovery rate for Cd-B was a mean of 102% (range: 89%-112%) at concentrations of 1, 10, and 15 μg/L (each with 3 samples). The within-day variation was 2.2% (range: 0.5%-3.7%), and the between-day variation was 3.1% (range: 2.4%-3.8%) at blood concentrations of 3.27 and 7.20 μg/L. These quality control data indicate the reliability of the measurement method used in this study. Future studies should consider refining measurement techniques and incorporating comprehensive exposure assessments to address these limitations.

## 5. Conclusions

This study examined the relationship between Cd-B and proximal tubular dysfunction among workers in Ni-Cd battery plants in Japan. The findings indicate that higher Cd-B levels observed among Japanese workers are associated with an increased risk of developing proximal tubular dysfunction, as measured by B2M-U. Whether or not the Cd-B cutoff value of 5 μg/L is adequate remains unclear. The significant risk of proximal tubular dysfunction in workers under current industrial occupational management in Japan warrants careful consideration. Even in developed countries such as Japan, it may be necessary to discuss and possibly change the existing exposure standards to improve workers' health.

## Data Availability

The data used in this study will be made available upon reasonable written request to the corresponding author.
